# The blossoming of methods and software in computational biology

**DOI:** 10.1371/journal.pcbi.1011390

**Published:** 2023-08-17

**Authors:** Feilim Mac Gabhann, Virginia E. Pitzer, Jason A. Papin

**Affiliations:** 1 Department of Biomedical Engineering and Institute for Computational Medicine, Johns Hopkins University, Baltimore, Maryland, United States of America; 2 Department of Epidemiology of Microbial Diseases and Public Health Modeling Unit, Yale School of Public Health, New Haven, Connecticut, United States of America; 3 Department of Biomedical Engineering, University of Virginia, Charlottesville, Virginia, United States of America

As we wrote previously [1], science benefits when we share not only our insights and discoveries but also the tools and approaches that we develop. This sharing improves reproducibility and reuse, and it enables others to build on our work. These tools and approaches are research accelerants and are the focus of 2 key sections in our journal: *Methods* and *Software*.

Since its founding in 2005, *PLOS Computational Biology* has been the home of exceptional computational research and cutting-edge methodological advances. We publish papers that use computational techniques to generate new biological insight, and we publish papers that describe software or methods that many other researchers in the field can use independently to generate new biological insight.

There is great merit in using existing techniques to advance biological understanding, and there is great merit in developing and sharing new techniques that will result in further advances. The field of Computational Biology supports both.

In 2013, the journal introduced the *Methods* section and the *Software* section to encourage more researchers to publish methodological advancements. From the inception of these sections, there were dedicated *Methods* Editors and *Software* Editors on the Editorial Board responsible for the papers submitted to each section. Through the end of 2022, those editors have guided the publication of 647 *Methods* papers and 286 *Software* papers, from among the 1,790 and 646 manuscripts submitted, respectively. The standard of those papers has been high, with many excellent and impactful papers published, covering methods for microbiome data analysis [2], deep learning to predict molecular interactions [3], and quantitative analysis of live-cell imaging [4], and software tools ranging from Bayesian Evolutionary Analysis [5] to multiomics integration and feature selection [6], and genome assembly [7].

At the outset, the concept of having separate *Methods*-specific and *Software*-specific editors was seen as beneficial to being able to set appropriate criteria for the review and publication of these papers, for example, making clear that new work that facilitated original research was publishable, even if the paper itself did not include original research findings. By creating and stewarding a clear standard for these papers, it has been possible to maintain and even increase the high standard of methods and software papers published in our journal.

**Fig 1:**
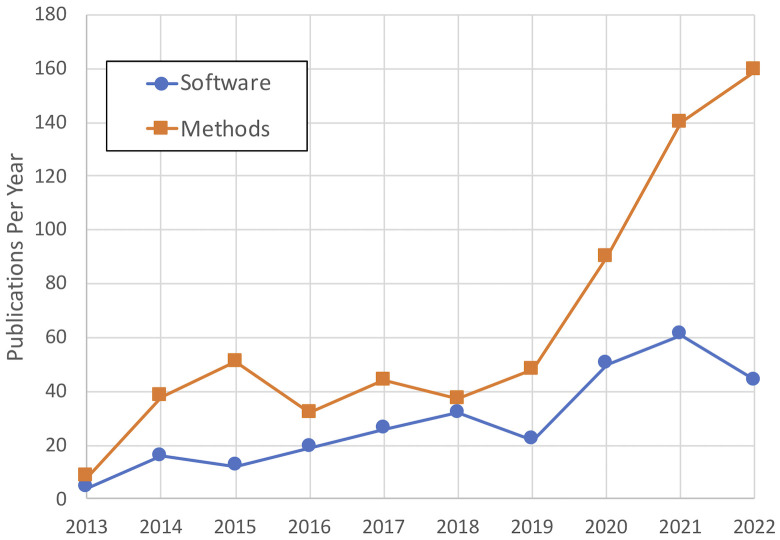
Number of *Methods* and *Software* papers published each year in *PLOS Computational Biology*, 2013–2022. The acceptance rates for submitted Methods and Software papers have been consistent at approximately 35% and 45%, respectively, for several years.

By all metrics, the *Methods* and *Software* sections have been a massive success and continue to grow. More than half of all submissions received since 2013 in these sections have come in the last 3 years (**Fig 1**). Our editors made a Herculean effort to meet this demand, but it became harder to keep up. Clearly, we needed to spread the handling of these papers across more people.

We also recognized that many, if not most, of our editors have expertise in methods development and/or software development in their respective fields. As Computational Biology and its subfields become more mature, the submissions we receive often are more specialized, describing computational techniques that are applied in specific subfields but not others. For example, techniques that are used to process neuroscience data may be quite different from those used to analyze genomic data. Thus, having expertise in those fields—as our Neuroscience and Genomics editors do—is increasingly helpful for the review and handling of these submissions.

Therefore, *Methods* and *Software* submissions are now handled by domain-specific editors, as other Research articles are, which both increases the pool of editors handling these submissions and provides each submission with editors that are familiar with the subtleties and background of the domain-specific approaches, applications, and implementations. This editorial structure also reflects the broadening reach of research and development into new methods and new software across various areas of Computational Biology. Authors will now designate which topical section of the journal their *Methods* or *Software* manuscripts are most suited to and suggest appropriate editors to handle their manuscript, as has been the case for other Research articles.

The development and use of tools and approaches are integral to modern science, and the exceptional and dedicated authors, editors, and reviewers have nurtured and grown the *Methods* and *Software* sections. In particular, we want to thank our *Methods* and *Software* Editors for their immense work over the years. They have built a great reputation for these sections and have set them on the path to continue to thrive. We also want to thank the Computational Biology community for submitting novel and creative work. There’s no doubt that *PLOS Computational Biology* has become a key venue for the sharing and advancement of novel tools and approaches.

We believe this new approach improves the handling of these papers and the experience of authors. *Methods* manuscripts and *Software* manuscripts will continue to be reviewed with the same criteria and standards as before; only now, the domain expertise of the editors will be more closely aligned to the manuscripts. *PLOS Computational Biology* will continue to be the home for sharing the development of excellent tools, approaches, and algorithms in our field.
